# Arteriovenous fistula of the mandible: a case report

**DOI:** 10.1007/s11282-024-00762-6

**Published:** 2024-06-19

**Authors:** Mattia Todaro, Ermal Pashaj, Alessandro Pedicelli, Andrea Alexandre, Gianmarco Saponaro, Giulio Gasparini, Alessandro Moro

**Affiliations:** 1 Maxillo-Facial Surgery Unit, IRCSS Fondazione Policlinico Universitario “A. Gemelli” – Università Cattolica del Sacro Cuore sede di Roma, 8 Largo Agostino Gemelli, 00168 Rome, Italy; 2 Centro Ospedaliero Universitario Catolico Nostra Signora del Buon Consiglio Rr. Dritan Hoxha, Tirana, Albania; 3 Radiology and Neuroradiology, Department of Diagnostic for Images, Oncological Radioterapy and Hematology, IRCSS Fondazione Policlinico Universitario “A. Gemelli” – Università Cattolica del Sacro Cuore sede di Roma, Largo A. Gemelli, 8, 00168 Rome, Italy

**Keywords:** Arteriovenous, Fistula, Oral and maxillofacial pathology, Dentigerous cyst, Vascular malformation, Embolization

## Abstract

Intraosseous arteriovenous malformations (AVM) are uncommon high-flow vascular malformation that can affect the maxilla or mandible. AVM may present with aspecific and misleading signs and symptoms. The diagnosis is often accidental and bleeding may represent the first symptom. Radiographically, there are few characteristic features and misdiagnosis is easy. Here we report the case of a young male affected by arteriovenous fistula on the right side of the mandible initially misdiagnosed as a cystic lesion. The patient underwent transarterial embolization of the vascular malformation and subsequently the lesion was surgically removed. 1-year follow-up showed complete healing of the mandibular bone and absence of recurrence. Intraosseous arteriovenous malformations are rare entities. However, due to their harmfulness, both clinicians and radiologists must be aware of this type of lesion and should always consider them in the differential diagnosis of osteolytic lesions.

## Introduction

In 1982, Mulliken and Young classified two types of vascular lesions based on their endothelial characteristics: haemangiomas and vascular malformations [1]. This classification was later adopted in 1996 by the International Society for the Study of Vascular Anomalies (ISSVA) and finally modified in 2018.

Haemangiomas are characterized by endothelial hyperplasia and cellular proliferation; they appear after birth and grow rapidly during the first months of life, then show involution over 5 or 6 years [2, 3].

The immunohistochemical marker GLUT-1 can be used to objectively differentiate hemangiomas (GLUT-1-positive) from vascular malformations (GLUT-1-negative) [4].

Vascular malformations derive from aberrations of vascular morphogenesis and are made by ectasic vessels, lined by flat endothelium, with no increased cellular turnover [4].

Vascular malformations can be subdivided based on the rate of blood flow: “slow flow” (capillary, venous, lymphatic or mixed) and “high flow” (arterial, arteriovenous, fistulae or shunt) subtypes (Table 1); approximately 51% occur in the head and neck.

**Table 1:** Vascular malformations classification by blood flow

High-flow	Arteriovenous malformation Arteriovenous fistula
Low-flow	Venous malformation Lymphatic malformation Lymphatic–venous malformation

Dental arcade arteriovenous fistula (DA-AVF) or intraosseous arteriovenous malformations (AVM) of the maxilla or mandible are rare [5]. AVFs are high-flow lesions characterized by congenital dysmorphogenesis of the arterial and venous structures of the dental arcade [6]. AVFs may present with many sneaky symptoms [7], such as gingival bleeding, slow-growing expansile masses and dental mobility, or sometimes bleeding may represent the first symptom: a biopsy or even a simple tooth extraction can cause life-threatening hemorrhage [8].

Due to the clinical and radiological similarities, the possibility of mistakenly diagnosing an AVF for a jaw cyst should always be considered [9]. In this case report, we describe an arteriovenous fistula of the mandibular body, initially diagnosed as an odontogenic cyst and we also describe the therapeutic path with special attention to the diagnostic strategies.

## Case report

A 24-year-old man was admitted to the maxillofacial surgery unit with a previous diagnosis of cystic lesions of the right-posterior mandible.

Medical examination revealed that the patient had subtotal blindness due to retinitis pigmentosa, no other abnormal clinical findings were found.

He had an unremarkable medical history with no previous trauma, infection, or surgery reported.

The extra-oral head and neck examination showed no anomalies.

Intraoral clinical examination revealed tooth mobility of the 46 and 47 tooth.

The dental pulp vitality test showed absence of vitality of elements 46–47–48 tooth.

There was no evidence of any alterations in the surface or color of the alveolar mucosa.

At the examination, no signs suggestive of vascular malformations were detected.

**Fig. 1:**
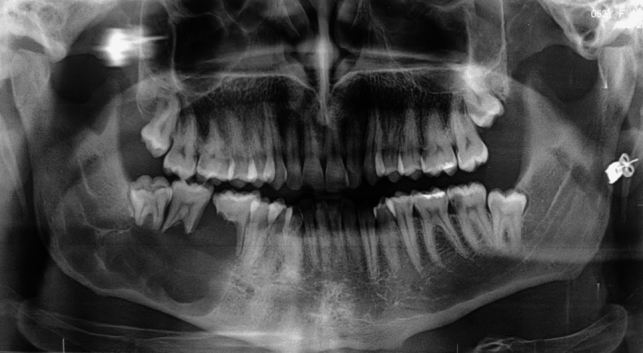
Panoramic radiography shows the lytic lesion in the right jaw

The panoramic radiography (Fig. 1) revealed a bone lesion located in correspondence to the dental elements 46–47–48, the lesion appeared radiolucent, multilocular, and extended from the 45 region to the right mandibular angle, severe roots resorption of the elements 46–47, and initial root erosion of element 4.8 was also observed.

**Fig. 2:**
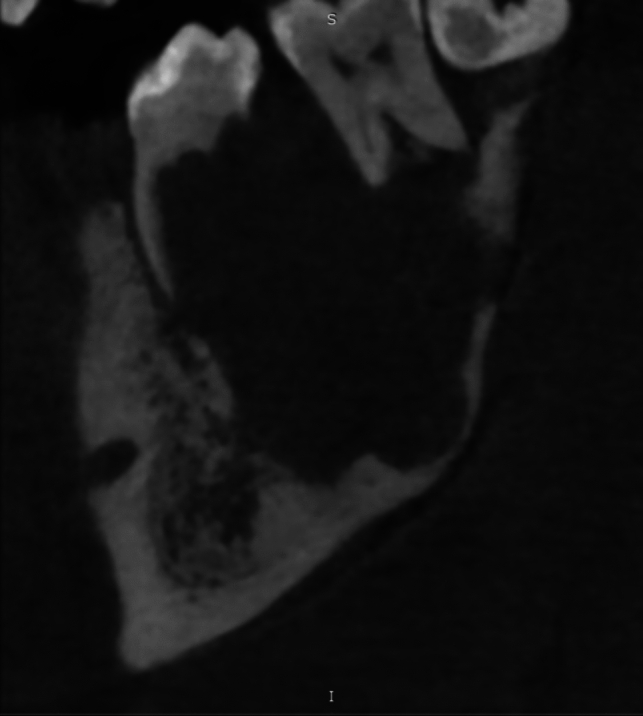
CT scan shows the osteolytic lesion and the erosion of the dental elements. In this image, the ragged aspect of root resorption is particularly evident

A CT scan of the lower jaw confirmed the presence of an osteolytic, expansile lesion extending from the right mandibular body to the right angle (Fig. 2).

The aforementioned area caused erosion of the upper part of mandibular vestibular cortical profile and disappearance of the trabecular pattern in the interdental space, with no significant changes in the deep cortical profile.

Based on the clinical features, a surgical enucleation with biopsy was planned.

The patient underwent surgical enucleation under general anesthesia and nasotracheal intubation. The mucoperiosteal flap was elevated and a vascular-like mass at the level of the upper vestibular cortex was noted as unexpected finding.

The lesion, purple, soft, and pulsatile was suggestive of a vascular malformation. On the base of these findings, the operation was stopped and the patient was transferred to the angiographic theater in order to perform a digital subtraction angiography (DSA) by trans-femoral approach.

**Fig. 3:**
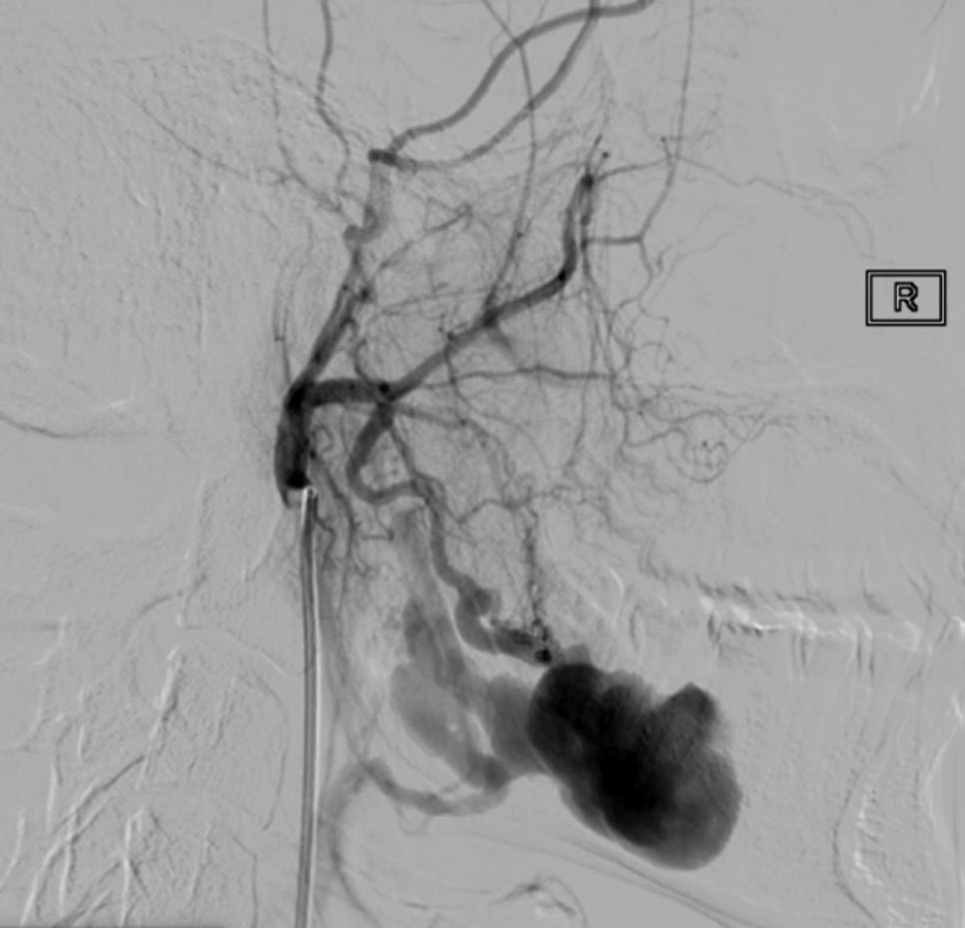
Selective angiography of the distal external carotid artery demonstrating the angioarchitecture of the arteriovenous fistula, arterial feeders, and the venous drainage into the right external jugular vein

DSA confirmed the suspicion of arteriovenous fistula (AVF) of the right mandibular body and angle (Fig. 3), with prevalent arterial feeders from the right inferior alveolar artery and secondary feeders from the ipsilateral ascending palatine artery and facial artery.

**Fig. 4:**
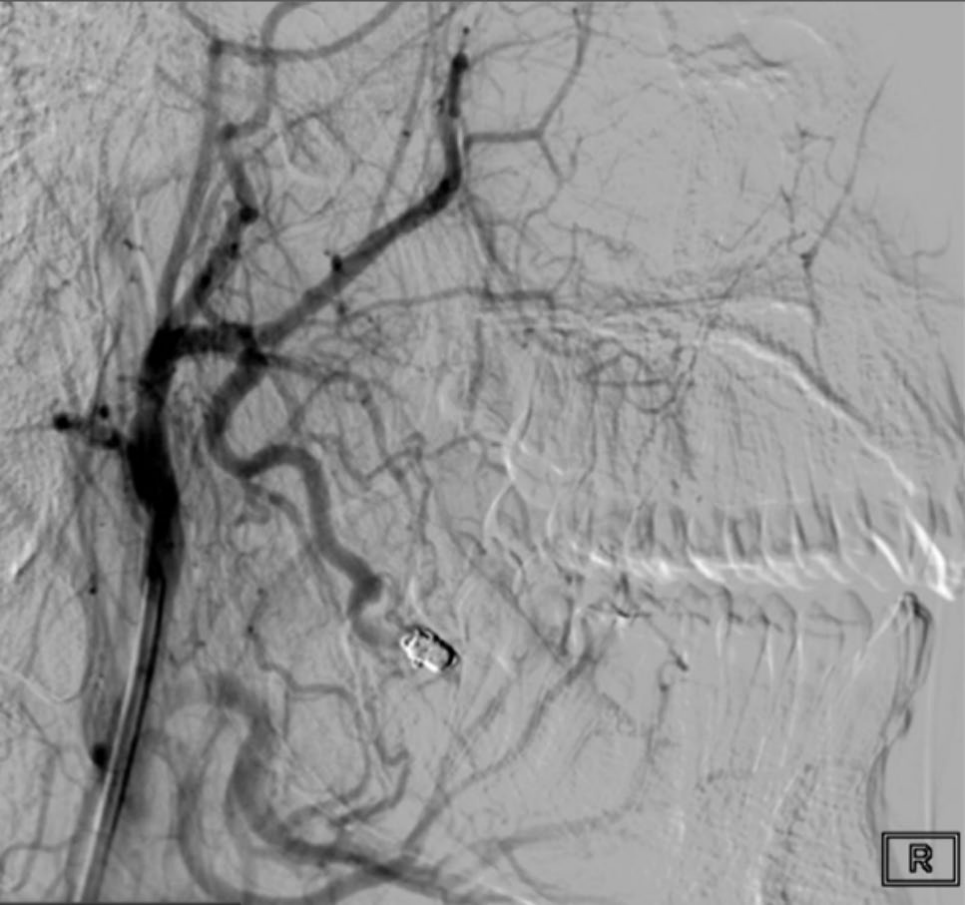
Embolization of the AVF with coils and the complete devascularization of the lesion

The fistulous pouch was located within the bone and it was draining through mandibular veins into the right external jugular vein. Superselective embolization was performed by placing three Guglielmi detachable coils (GDC) at the shunting point of the AVF, using a microcatheter Headway Duo 17 (Microvention Inc.- CA, USA) with no procedural complications. The occlusion was confirmed by post-embolization angiography (Fig. 4).

After two days, the patient underwent surgical re-intervention under general anesthesia. The lesion was enucleated and 46–47–48 tooth were extracted with no intraoperative complications.

The histopathological examination confirmed the vascular nature of the lesion, in particular the histological analysis pointed out the presence of a de-epithelized formation with fibroconnective wall, with deposits of hemosiderin, containing fibrinohematic material. There was no presence of cellular atypia.

The patient-reported paresthesia of the lower lip completely recovered after 8 months.

**Fig. 5:**
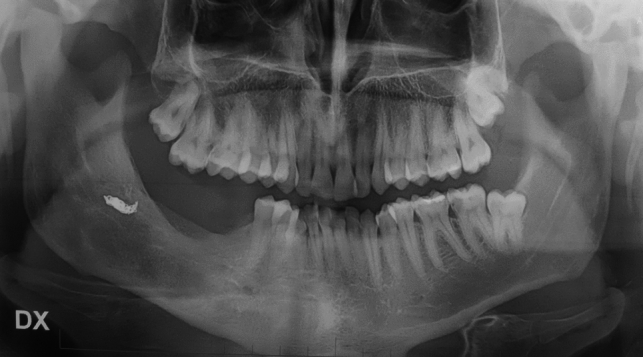
The 1-year post-operatory panoramic radiographic

**Fig. 6:**
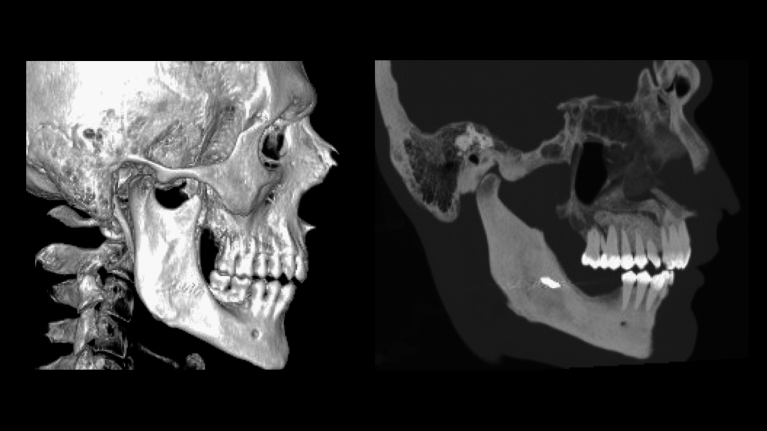
One year after the intervention, the CT showed complete resolution of the AVM and complete ossification of the right body of the mandible

**Fig. 7:**
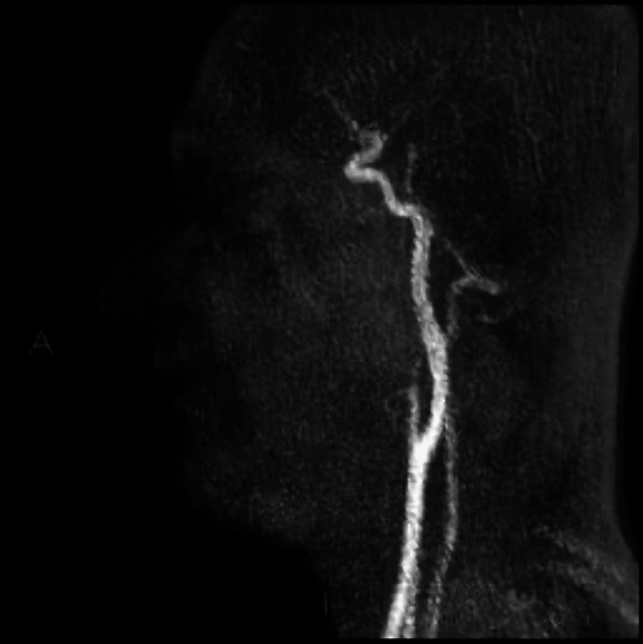
One year after embolization and intervention, the magnetic resonance angiography showed complete resolution of the AVM

The panoramic radiographic (Fig. 5) and the computed tomography (Fig. 6) performed 1 year after the treatment showed a complete reossification of the right mandible and the magnetic resonance angiography (Fig. 7) showed complete resolution of the AVM.

## Discussion

AVFs are vascular malformations characterized by the unusual shunt between arteries and veins, often enlarged; the hematic blood flow bypasses the capillary bed, so the local pressure is not downregulated. AVFs of the mandible can be acquired or congenital. The acquired forms are usually caused by a trauma involving the jaws. Congenital forms are present at birth and enlarge with physical growth; the growth is due to hormonal factors, vasomotor disturbances, or trauma. These DA-AVFs may be also a presentation of cerebrofacial arteriovenous metameric syndrome (CAMS).

Clinical and radiographic features of these lesions help in establishing a differential diagnosis. Despite this fact, signs and symptoms are often not univocal. A dental AVF is difficult to differentiate on the basis of their radiographic features alone and the misdiagnosis is easy.

These lesions are usually present at birth and enlarge with the growth of the child; there is generally no spontaneous regression. To our knowledge, only one case of regression after incisional biopsy and diagnostic angiography is reported [10].

A gradual increase in dimension is usually reported [11]. These lesions may also develop after penetrating trauma or blunt injury [12, 13].

The clinical characteristics of high-flow vascular malformations of the jaws are variable. Although infrequent, in cases with soft tissue involvement, typical symptoms like audible bruit, palpable thrill, pulsatile swelling, or the presence of vascular nevi and dilated veins may be found and must immediately grab the clinician's attention toward the possible presence of a vascular malformation.

The involvement of soft tissue seems to be more frequent in maxillary vascular malformations than in mandibular ones.

Other less specific clinical features are teeth loosening, dental misalignment, gingival bleeding, and neurosensory deficits [14]. Near-fatal hemorrhage is in some cases the first clinical sign of their presence [15].

The diagnosis is based on a combination of clinical and radiological features. There is a wide range of imaging techniques, including plain radiography, Doppler ultrasonography, computed tomography angiography (CTA), magnetic resonance imaging (MRI), magnetic resonance angiography (MRA), and conventional angiography.

The first radiological diagnosis of jaw lesions usually relies on panoramic radiography.

On plain radiographs, vascular malformations appear cystlike lesions and unfortunately, there is often a lack of unique and distinctive signs.

Characteristics that should be considered suspicious for vascular malformations are: “soap bubble” or “honeycomb” aspect of the radiolucencies and resorption of roots.

Another characteristic sign is the presence of an expanded or double mandibular channel. This bony expansion is due to the intraosseous dilatation of the inferior alveolar artery and vein [16], and some authors also reported the evidence of an irregular widening of the inferior alveolar canal [17].

The lack of a discrete margin and of a sclerotic rim can help differentiate these lesions from dentigerous cysts.

The margins may also appear erosive, thus simulating a malignant lesion [18].

Roots resorption is another noticeable feature as reported by Darcey et al. [19] in case of slowly expanding lesions of the jaws. The roots resorptive pattern is usually focal and well-defined; on the contrary, in case of malignancies, the resorption pattern is often “ragged”; in this case, the pattern is particularly irregular and notched.

The cortical bone can be perforated over the expanded area, and the involvement of adjacent soft tissues may be present [3, 20].

Ultrasonography may be used to detect ecstatic vessels with high flow and low resistance Doppler signals but is not usually considered.

CT angiography is a valid option. It shows the vascular lesion and can help identify both the location and contributory feeding vessels.

MRI/MRA detect flow voids and can help distinguish between tumors and malformations.

Angiography is the gold standard to confirm the diagnosis and a fundamental tool to understand the architecture and the features of these lesions.

In our case, the radiographic and clinical appearance was comparable with that of a large odontogenic keratocyst or ameloblastoma.

Therapeutic ways include endovascular treatments (transarterial, transvenous, or percutaneous embolization), surgery, or a combination of those two.

Ligation of the external carotid artery has been previously described as a purely symptomatic treatment, before surgery, or as an emergency procedure.

Nowadays, there is little or no place for this procedure since it is well established that collateral circulation will rapidly be recruited from either the contralateral external carotid or vertebral systems. Moreover, the ligation of the external carotid artery may prevent any future embolization [21, 22].

Endovascular treatment is considered the first-line treatment for high-flow vascular malformations of the jaws. The literature shows a success rate of more than 70% [9].

A large variety of coils and embolic agents may be used for AVF embolization. The choice is subordinate to lesion’s site, size, and morphology.

Direct transosseous embolization may be evaluated for complex, high-flow lesions in which venous pouches are not accessible by transarterial or transvenous route [10, 23].

Embolization, however, is not a procedure free from complications; serious drawbacks like occlusion of pulmonary or cerebral vessels or recurrence of the vascular malformation should be considered.

Other reported problems of embolization include blindness, infection, tissue necrosis, severe pain, and poor cosmetic outcome.

To avoid revascularization, in particular in case of subtotal occlusion, surgery is usually indicated after endovascular embolization [24].

The timing of surgery remains a point of discussion. We believe that to avoid revascularization of the lesion, surgical enucleation should always be performed after embolization. The interval between the procedures should include between 48 h and 2 weeks. After the mere embolizations, bone remineralization should occur and according to Chhoeurn et al. should be completed within two years [25].

In our opinion, whenever feasible, the surgical removal of the embolized lesion should be performed since this is the only way to guarantee the complete reossification of the involved bone segment, thus providing a complete anatomical and functional recovery.

High-flow vascular lesions of the jaws are rare entities, but clinicians should always consider them in the differential diagnosis of osteolytic lesions of the jaws.

It is important to keep in mind the hemorrhagic potential of these lesions: a simple procedure, like an extraction, in the presence of an arteriovenous fistula may result in severe, potentially lethal, hemorrhage.

When detected, DSA and pre-operative embolization is a fundamental resource in the management of these lesions.

## Data Availability

No data set was generated for this article.
